# Supplementary figures and images for: Patterns of mental health problems before and after easing COVID-19 restrictions: Evidence from a 105248-subject survey in general population in China
Source: PLoS One. 2021 Aug 3;16(8):e0255251. doi: 10.1371/journal.pone.0255251 (PMC8331222; doi:10.1371/journal.pone.0255251)

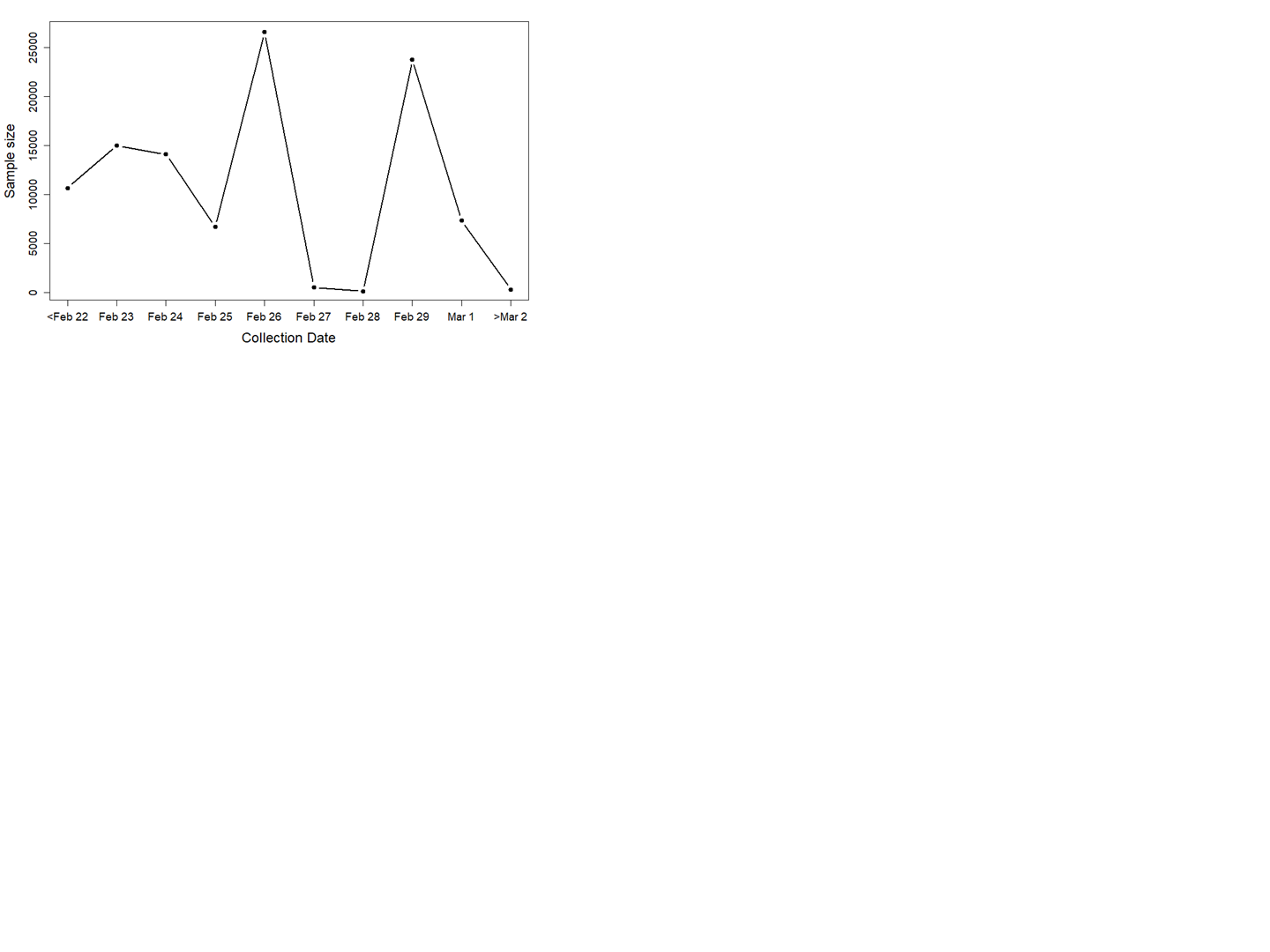

Supplement: S1 Fig — (TIF) [file pone.0255251.s001.tif]
