# Supplementary material for: Patterns of mental health problems before and after easing COVID-19 restrictions: Evidence from a 105248-subject survey in general population in China
Source: PLoS One. 2021 Aug 3;16(8):e0255251. doi: 10.1371/journal.pone.0255251 (PMC8331222; doi:10.1371/journal.pone.0255251)
Supplement: S1 Table — (DOCX) [file pone.0255251.s002.docx]

S1 Table. Sample Characteristics of Survey Participants Before and After Easing Restrictions

|  | Before Easing Restrictions (N=46508) | After Easing Restrictions (N=58740) |
| --- | --- | --- |
| Gender |  |  |
| Female | 41.3% | 49.0% |
| Male | 58.7% | 51.0% |
| Marriage status |  |  |
| Married | 49.6% | 59.5% |
| Divorced/Widowed | 48.6% | 38.3% |
| Single or never married | 1.8% | 2.2% |
| Education |  |  |
| High school or below | 36.3% | 41.5% |
| University/college or above | 63.7% | 58.5% |
| Occupation |  |  |
| HWs^a^ | 16.7% | 14.1% |
| Non-HWs | 66.5% | 65.6% |
| Unemployed | 16.8% | 20.3% |
| Disease status |  |  |
| No | 96.2% | 97.2% |
| Respiratory^b^ | 0.2% | 0.2% |
| Non-respiratory^c^ | 2.8% | 2.2% |
| Both | 0.8% | 0.4% |
| Perceived needs met |  |  |
| Yes | 95.6% | 96.6% |
| No | 4.4% | 3.4% |
| Age (mean, std) | 28.7(9.4) | 31.0(9.9) |

Note:

^a^ Healthcare workers including doctors, nursing professionals, midwifery professionals, dentists and pharmacists;

^b^ Respiratory diseases including pneumonia, asthma, and COPD;

^c^ Non-respiratory disease including hypertension, diabetes, heart disease, stroke, hepatitis, cancer, or esophagitis, gastritis, or duodenitis or other.
